# Insulinoma: Metastatic Recurrence 38 Years Following Initial Diagnosis in Pregnancy

**DOI:** 10.1210/jcemcr/luad168

**Published:** 2024-01-08

**Authors:** Christopher A Preston, Nirupa Sachithanandan, Ie-Wen Sim, Jon van Heerden, Stephen Farrell

**Affiliations:** Department of Endocrinology & Diabetes, St Vincent’s Hospital Melbourne, Fitzroy, Victoria 3065, Australia; Department of Endocrinology & Diabetes, Western Health, Melbourne, Victoria 3021, Australia; Department of Endocrinology & Diabetes, St Vincent’s Hospital Melbourne, Fitzroy, Victoria 3065, Australia; Department of Medicine, The University of Melbourne, Fitzroy, Victoria 3065, Australia; Department of Endocrinology & Diabetes, St Vincent’s Hospital Melbourne, Fitzroy, Victoria 3065, Australia; Department of Endocrinology & Diabetes, Western Health, Melbourne, Victoria 3021, Australia; Department of Medicine, The University of Melbourne, Fitzroy, Victoria 3065, Australia; Department of Endocrinology & Diabetes, Monash Health and Eastern Health, Melbourne, Victoria 3168, Australia; Department of Medicine, Monash University, Clayton, Victoria 3168, Australia; Department of Surgery, Mayo Medical School, Rochester, MN 55905, USA; Department of Surgery, Mayo Medical School, Mayo Clinic, Rochester, MN 55905, USA; Department of Surgery, Medical University of South Carolina, Charleston 29425, USA; Department of Surgery, St Vincent’s Hospital Melbourne, Fitzroy, Victoria 3065, Australia; Department of Surgery, Austin Health, Heidelberg, Victoria 3084, Australia

**Keywords:** insulinoma, recurrence, pregnancy, Ga-68 GLP-1 PET

## Abstract

A case of recurrent insulinoma spanning 4 decades is described. Following a delayed diagnosis, hyperinsulinemic hypoglycemia was confirmed in a 24-year-old woman during early pregnancy. Initial surgery, culminating in subtotal pancreatectomy, was noncurative. A 1-cm insulinoma was subsequently resected from the head of the pancreas postpartum, with postoperative resolution of hypoglycemia. However, 32 years later, the patient experienced a recurrence of hypoglycemic symptoms. Eventually, a subcentimeter extrapancreatic lesion was identified anterior to the pancreatic head on gallium-68 DOTA-Exendin-4 positron emission tomography/computed tomography. In 2022, a third operation was performed, with excision of a 4 × 3 mm tumor adjacent to the pancreatic head, and histology confirming insulinoma. She was again cured of symptoms.

## Introduction

Insulinomas are rare neuroendocrine tumors. Most are sporadic, intrapancreatic, benign, and curable, while a minority are malignant or associated with multifocal lesions in hereditary syndromes [1‐3]. Presentation rarely occurs during pregnancy. Recurrence may be associated with incomplete resection, malignancy with local, regional, or distant metastases, and occasionally a second, separate intrapancreatic tumor. Rarely does the condition recur after a prolonged delay.

Here, we report a case of insulinoma diagnosed in pregnancy, undergoing initial failed surgery, then successful resection 6 weeks postpartum. Remarkably, a local recurrence was diagnosed 32 years later. This was again successfully resected after many years’ delay. There is no other history of personal or familial endocrinopathies.

## Case Presentation

The patient, a 24-year-old mother, was diagnosed with a presumed insulinoma in September 1984. Six months earlier, she had developed symptoms including frequent episodes of confusion, diplopia, and occasional blackouts. These typically occurred at night, especially following physical exertion. She also experienced profuse sweating overnight and was sometimes difficult to rouse. Between episodes, she was increasingly confused and vague. Drinking juice or eating snacks helped alleviate her symptoms.

She was initially misdiagnosed by several doctors and considered to have a primary seizure disorder. She underwent multiple investigations, including electroencephalography and neuroimaging, and she was commenced on antiepileptic medication. After several months of assessments and much associated distress, her neurologist eventually performed a random blood glucose assessment, which revealed a blood glucose level of 2.0 mmol/L (36 mg/dL).

At this stage, in September 1984, she was admitted to a tertiary referral hospital for a 72-hour fast. Within hours, she developed neuroglycopenic symptoms, with a blood glucose level of 1.2 mmol/L (21.6 mg/dL), in the context of inappropriately elevated insulin at 80 mU/L (normal fasting <10) and C-peptide at 5.60 pmol/mL (0.18-0.63). A sulfonylurea screen was negative. Abdominal ultrasound and noncontrast computed tomography (CT) of the pancreas did not demonstrate any mass lesion. Venous sampling was not performed, as by this stage she was 12 weeks pregnant.

One week later she proceeded to open abdominal surgery under a general surgery team. Full exploration for a pancreatic tumor failed to reveal any abnormality. She proceeded to “blind” subtotal pancreatectomy, as was the prevailing Australian approach at that time for surgically nonlocalized disease; approximately 75% of the was pancreas removed, and the operation was complicated by splenectomy. Histology was normal with no evidence of insulinoma or nesidioblastosis.

The surgery did not add significantly to her morbidity. She recovered well; however, she continued to suffer from hypoglycemic episodes, requiring frequent carbohydrate-containing meals, including overnight. She was discharged home and her symptoms improved considerably throughout her pregnancy. She went on to deliver a healthy baby at full term, who did not suffer any dysglycemia.

Unfortunately, 7 days postpartum, her symptoms recurred with an associated nocturnal seizure.

Repeat imaging with contrast-enhanced CT and endoscopic ultrasound was unremarkable, as was celiac angiography. Oral diazoxide was commenced and the dose progressively increased to 100 mg 3 times daily to maintain normoglycemia, although she still required overnight feeds. At this dose, she experienced the adverse effects of ankle and periorbital fluid retention, requiring loop diuretics with potassium supplementation. She was then referred to the Mayo Clinic, where a repeat fast in May 1985 was again terminated early due to unresponsiveness at a blood glucose level of 2.3 mmol/L (41.4 mg/dL) with an insulin level of 13 mU/L. Repeat ultrasound and CT were again unremarkable.

**Figure 1.**
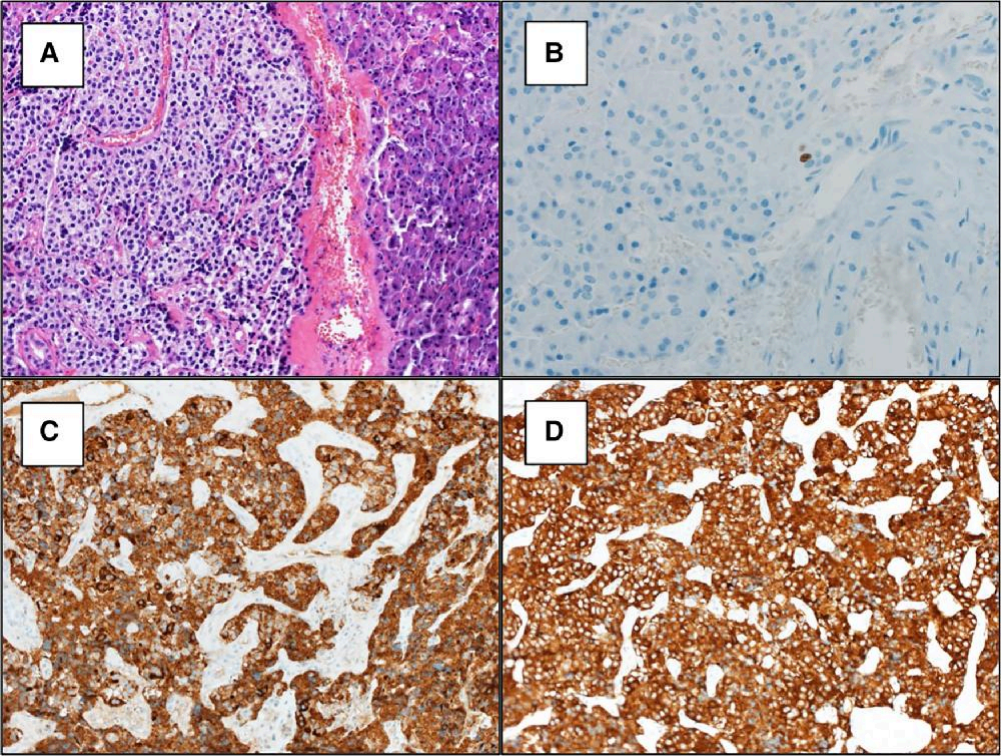
(A) Histopathology of 1-cm tumor resected from the head of the pancreas in May 1985 demonstrating a well-circumscribed insulinoma. (B) Ki67 index of approximately 1%. C. Chromogranin A staining. D. Synaptophysin staining.

She proceeded to repeat open abdominal surgery, with intraoperative bimanual palpation and ultrasound examination of the remnant pancreas, localizing a 1-cm nodule within the pancreatic head, 5 mm from the line of previous pancreatectomy. An enucleation of the insulinoma was performed, with immediate resolution of hypoglycemia. Histology confirmed a well-differentiated neuroendocrine tumor, which stained strongly positive for synaptophysin and chromogranin A, with a Ki67 index of approximately 1%, and no features of malignancy (Fig. 1). Postoperatively, she ceased diazoxide and remained well, with no further hypoglycemia for many years.

However, 32 years later, in 2017, her symptoms recurred, again characterized by episodic confusion, diplopia, and sweating, and again most pronounced overnight.

Initially, due to fear of hypoglycemia, the patient self-managed the recurrence with increased oral intake, resulting in significant weight gain. In 2018, a repeat fast was aborted early by the patient at a glucose of 2.6 mmol/L (46.8 mg/dL), with insulin and C-peptide suppressed at 2.0 mU/L and 0.56 pmol/L respectively. Magnetic resonance imaging (MRI) of the pancreas and gallium-68 (Ga-68) DOTATATE–positron emission tomography (PET) were unable to localize any definite insulinoma recurrence.

**Figure 2.**
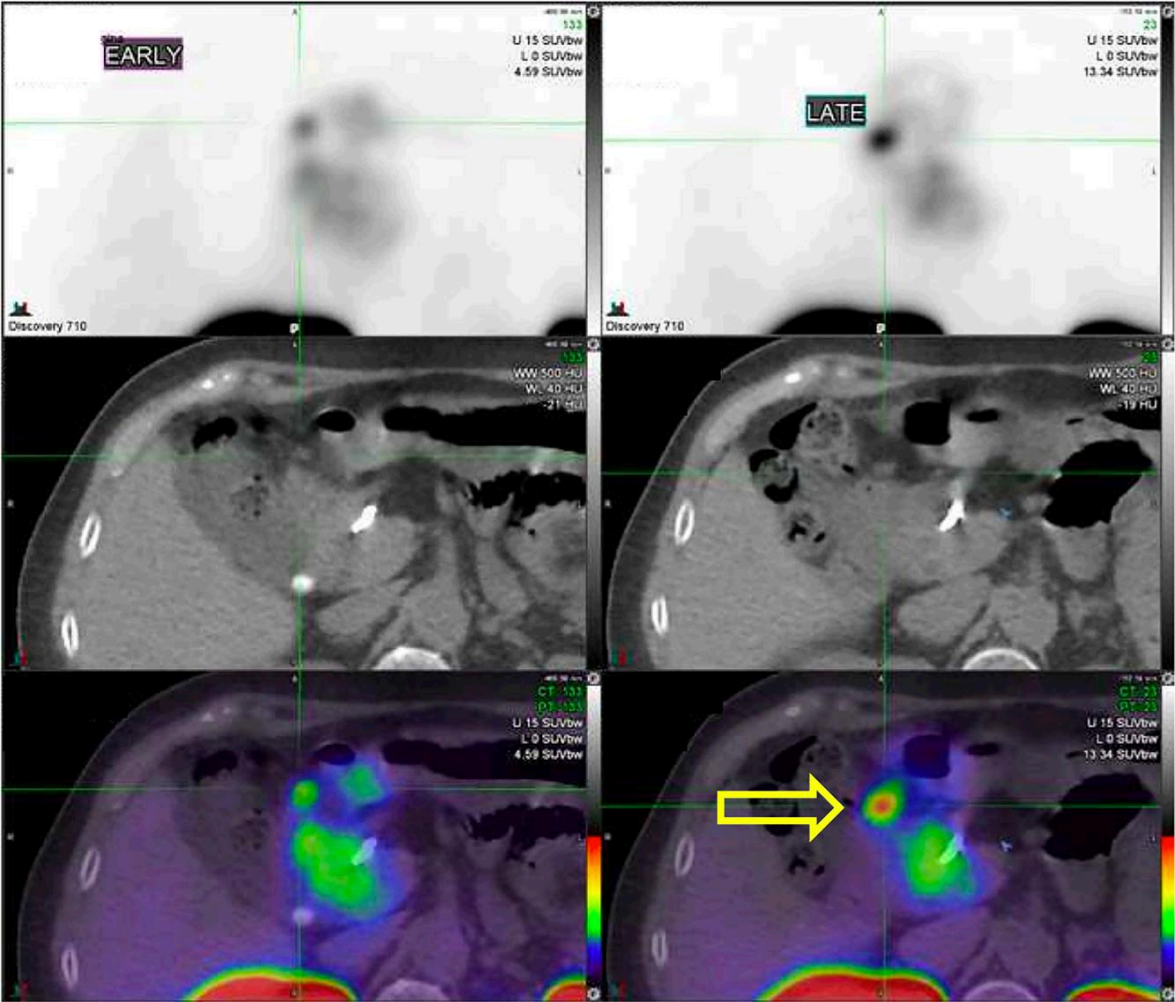
Apparent localization of insulinoma (arrow) with Ga-68 GLP-1 PET-CT.

She remained symptomatic, and subsequent fasting bloods demonstrated inappropriately elevated insulin of 9.0 mU/L and proinsulin of 16.0 pmol/L (<13.3) at a glucose of 2.4 mmol/L (43.2 mg/dL). Following pandemic-related delays, she eventually underwent further investigation with endoscopic ultrasound and noncontrast MRI, which were both unable to localize a lesion. A Ga-68 DOTA-Exendin-4 (Ga-68 GLP-1 PET) was performed and demonstrated intense uptake in a very small lesion anterior to the pancreatic head, consistent with an ectopic or metastatic pancreatic insulinoma (Fig. 2). This was not visible on repeat CT or contrast-enhanced MRI.

**Figure 3.**
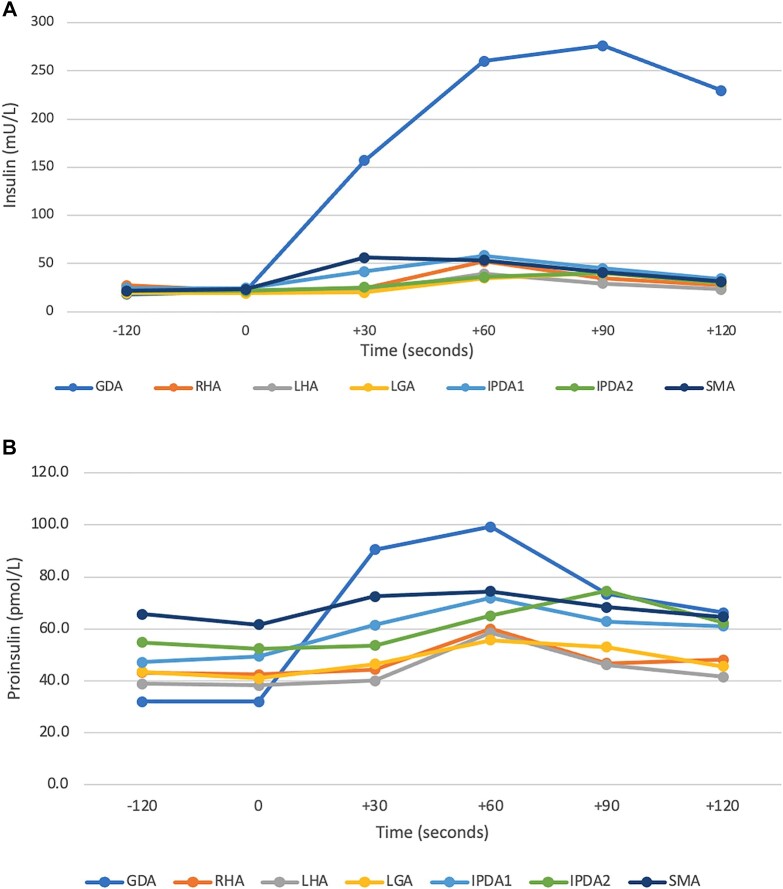
Calcium stimulation test demonstrating a maximal rise in insulin (A) and proinsulin (B) following calcium injection into the gastroduodenal artery. Abbreviations: GDA, gastroduodenal artery; RHA, right hepatic artery; LHA, left hepatic artery; LGA, left gastric artery; IPDA1, inferior pancreaticoduodenal artery, branch 1; IPDA2, inferior pancreaticoduodenal artery, branch 2; SMA, superior mesenteric artery.

In September 2022, another fast was terminated after 30 hours at a glucose of 2.0 mmol/L (36.0 mg/dL), with an insulin level of 8.0 mU/L and C-peptide of 0.63 pmol/mL. A calcium stimulation test demonstrated a maximal rise in insulin and proinsulin after calcium injection into the gastroduodenal artery, suggesting a lesion in or near the pancreatic head, concordant with the findings on Ga-68 GLP-1 PET (Fig. 3).

In November 2022, she proceeded to a third open surgery, guided by the localization studies, with successful excision of a small extrapancreatic tumor from just anterior to and above the pancreatic head, near the gastroduodenal junction. Histology confirmed a well-differentiated insulinoma, which again stained strongly positive for synaptophysin and chromogranin A, with a Ki67 index of 3%. There was no associated residual lymph node or pancreatic tissue. It is consistent with a local metastasis.

Postoperatively, she has remained well with no further hypoglycemia.

## Diagnostic Assessment

In 1984, abdominal ultrasound, noncontrast CT, and an intravenous glucose tolerance test were nonlocalizing. In 1985, contrast-enhanced CT, celiac angiography, and endoscopic ultrasound were also nonlocalizing, as were ultrasound pancreas and liver, and repeat CT. Intraoperative ultrasound localized a 1-cm nodule within the pancreatic head.

In 2018, MRI pancreas and Ga-68 DOTATATE-PET were unremarkable. In 2020, endoscopic ultrasound was unrevealing, as was subsequent noncontrast MRI. In 2022, Ga-68 GLP-1 PET localized a lesion anterior to the pancreatic head, which was not apparent on CT or contrast-enhanced MRI but was concordant with a subsequent calcium stimulation test.

## Treatment

From 1984 to 1985 and from 2017 to 2022, treatment included regular carbohydrate-containing meals and oral diazoxide with varying doses up to 100 mg 3 times daily.

In September 1984, open abdominal exploration and subtotal pancreatectomy was unsuccessful. In May 1985, repeat open surgery with intraoperative ultrasound facilitated successful enucleation of insulinoma from the pancreatic head.

In November 2022, open pancreatic surgery was again performed, with physical and ultrasound examination of the remnant pancreas and surrounds, and successful excision of ectopic insulinoma.

## Outcome and Follow-Up

The patient is now 63 years of age and has been followed from 1984 to the present. She keeps busy as a mother to 5 adult children and a grandmother to 14 grandchildren, who are all medically well. She remains physically active and has not experienced any further hypoglycemia following her most recent surgery, nor has she developed diabetes (HbA1c 5.6%) after so many pancreatic operations. Her weight has fluctuated from 73 kg in 1984 to a peak of 82 kg in 2022, with her most recent weight 68 kg with a body mass index of 25.9 kg/m^2^.

Apart from the requirement for splenectomy during her first surgery, her main adverse effect from treatment has been periorbital and peripheral edema following diazoxide use, which was controlled with medications. She also experienced a reaction to iodine contrast during her initial work-up, necessitating caution with subsequent imaging.

At last review, she was referred for consideration of genetic testing.

## Discussion

Insulinomas are rare and symptoms are usually initially misdiagnosed, contributing to delays in diagnosis and treatment [1, 4]. While most occur sporadically as solitary benign lesions, a minority are associated with inherited disorders, and fewer still are malignant. Insulinoma recurrence rates outside of malignant disease are low, estimated at less than 5%, and even lower in patients without co-existing multiple endocrine neoplasia, with most nonmalignant recurrences manifesting as small (<2 cm) intrapancreatic lesions, rather than the larger, often extrapancreatic lesions seen in malignant disease [5‐7]. To our knowledge, this case, extraordinarily, presents by far the longest reported latency period to recurrence.

In this case, whether the initial successful surgery was enucleation or more extensive re-resection, the surgery would be considered definitive. We assume there was a micro-metastasis, present prior to resection. Local recurrence within the pancreas may be attributed to, and is a risk of, enucleation, but regional metastasis is considered equally likely, regardless of resection technique.

Initial biochemical assessment in the work-up of hypoglycemia includes fasting plasma glucose paired with insulin and C-peptide levels, β-hydroxybutyrate, and screening for oral hypoglycemic agents. Fasting proinsulin levels may also be helpful, as a minority of pancreatic neuroendocrine tumors preferentially secrete proinsulin over insulin or co-secrete both [8].

Pregnancy in general is a time of maternal immunotolerance and insulin resistance, and endocrinologists should be aware of the expected improvement in hyperinsulinemic hypoglycemia with gestation, as was the case in our patient. There is also a corresponding risk of disease flare postpartum, so close clinical monitoring at that time is certainly advisable.

Our case also highlights the progress that has been made in localization studies for insulinomas over the past few decades. At the Mayo Clinic, in 1985, our case benefited from early expertise in the use of intraoperative ultrasound—now one of the mainstays of surgical assessment. Unlike other pancreatic neuroendocrine tumors, in which somatostatin receptors are usually overexpressed, insulinomas typically overexpress glucagon-like peptide-1 (GLP-1) receptors, making Ga-68 GLP-1 PET highly sensitive for the identification and localization of these tumors [9]. In the recent episode, it is worth noting the usefulness of Ga-68 GLP-1 PET, as the only imaging study which was positive in this tiny tumor. Calcium stimulation testing, which has superseded portal venous sampling, also helped by confirming that the hypersecretion of insulin and proinsulin was originating in or near the pancreatic head [10].

While pregnancy remains a relative contraindication to many imaging modalities requiring radiation or intravenous contrast, the risk-vs-benefit of Ga-68 GLP-1 PET, as the single most useful test, may be considered, if all other non-radiating investigations have failed.

While our patient had no personal or family history of other endocrinopathies associated with inherited endocrine disorders, genetic testing has been recommended, and it would be interesting to confirm the presence or absence of any mutations. However, as she has not had hyperparathyroidism, she is unlikely to have MEN1. Referral to a geneticist has been arranged and the outcome of testing is awaited.

Finally, our case also exemplifies the utility of ongoing follow-up following surgical cure of insulinoma. Certainly, a low threshold is warranted to investigate recurrent hypoglycemic symptoms in such patients, even if the initial diagnosis was one of benign insulinoma.

## Learning Points

- Insulinomas are rare and despite typical symptoms are often initially misdiagnosed. These patients should be managed in centers with expertise in insulinoma.
- Symptoms of hypoglycemia may improve during pregnancy and flare postpartum.
- Ga-68 GLP-1 PET should be considered for occult insulinoma localization.
- Calcium stimulation testing has a role in “functional regionalization” of the source of excess insulin.
- Genetic testing should be considered in young patients, in those with hyperparathyroidism, pituitary tumor, multiple pancreatic tumors, those with recurrence, and those with a family history of endocrine tumors.

## Data Availability

Data sharing is not applicable to this article as no datasets were generated or analyzed during the current study.

## Abbreviations

CT: computed tomography
Ga-68: gallium-68
GLP-1: glucagon-like peptide-1
MRI: magnetic resonance imaging
PET: positron emission tomography

## References

[1] Service FJ, Mcmahon MM, O’brien PC, Ballard DJ. Functioning insulinoma—incidence, recurrence and long-term survival of patients: a 60-year study. Mayo Clin Proc. 1991;66(7):711‐719. 1677058 10.1016/s0025-6196(12)62083-7

[2] Okabayashi T, Recio-Cordova JM. Diagnosis and management of insulinoma. World J Gastroenterol. 2013;19(6):829‐873. 23430217 10.3748/wjg.v19.i6.829 PMC3574879

[3] Baudin E, Caron P, Lombard-Bohas C, et al Malignant insulinoma: recommendations for characterization and treatment. Ann Endocrinol (Paris). 2013;74:523‐533. 23993836 10.1016/j.ando.2013.07.001

[4] Anderson CW, Bennett JJ. Clinical presentation and diagnosis of pancreatic neuroendocrine tumors. Surg Oncol Clin N Am. 2016;25(2):363‐374. 27013370 10.1016/j.soc.2015.12.003

[5] Yu J, Ping F, Zhang H, et al Clinical management of malignant insulinoma: a single institution’s experience over three decades. BMC Endocr Disord. 2018;18(1):92. 30522468 10.1186/s12902-018-0321-8 PMC6282250

[6] Gonzalez-Gonzalez A, Recio-Cordova JM. Liver metastases 9 years after removal of a malignant insulinoma which was initially considered benign. JOP. 2006;7(2):226‐229. 16525209

[7] Sheikh A, Zuberi L, Haque N. Rare among the rarities—recurrent insulinoma. J Coll Physicians Pak. 2007;17(6):364‐366. 17623590

[8] Murtha TD, Lupsa BC, Majumdar S, Jain D, Salem RR. A systematic review of proinsulin-secreting pancreatic neuroendocrine tumors. J Gastrointest Surg. 2017;21(8):1335‐1341. 28510792 10.1007/s11605-017-3428-8

[9] Falconi M, Eriksson B, Kaltsas G, et al ENETS consensus guidelines update for the management of patients with functional pancreatic neuroendocrine tumors and non-functional pancreatic neuroendocrine tumors. Neuroendocrinology. 2016;103(2):135‐171. 10.1159/000443171 PMC4849884 26742109

[10] Graf A, Sarlos S, Farrell SG, MacIsaac RJ, Inder WJ, Sachithanandan N. Selective intra-arterial calcium stimulation test for the localization of insulinomas: an Australian hospital experience. ANZ J Surg. 2020;90;E172‐E176. 32356594 10.1111/ans.15913

